# Algoman: Gearing up for the “Net Generation” and Era of Artificial Intelligence, One Step at a Time

**DOI:** 10.1007/s12098-019-03073-5

**Published:** 2019-10-16

**Authors:** Satish Deopujari, Ayush Shrivastava, Akruti Gore Joshi, Ashwin Meshram, Shashikant Chaudhary

**Affiliations:** 1 Department of Pediatrics, Nelson Mother & Child Care Hospital, Dhantoli, Nagpur, Maharashtra India; 2 Department of Pediatrics, Jawaharlal Nehru Medical College, Datta Meghe Institute of Medical Sciences (Deemed to be University), Sawangi, Meghe, Wardha, Maharashtra India; 3 Nucleus Children Hospital, Dhantoli, Nagpur, Maharashtra India; 4 Spinnaker LLC, Mumbai, India; 5 Cofounder Nagpur Angels, Ex-managing Director Global Logic, Nagpur, India

Apps in medicine are based on algorithms and are getting increasingly used, specially in critical care [1, 2]. Some of these apps are time sensitive and thus have given rise to the concept of “golden minute” and have helped reduce mortality. Unfortunately, these algorithms are still not very easily available at bedside, specially on devices like mobile phones. In contrast to its competitors *e.g*., Algomed, Algoman allows the user to create algorithms.

There is no denying that there is a e-revolution in medical education and practice [3]. The era of artificial intelligence in medicine is approaching fast [4]. ALGOMAN is a timely intervention to gear up the “net generation” of medical students, medical educators and clinicians.

Algoman creates algorithms that turn into an app on the smartphones that can act like “pocket brains”. This waives off the need to navigate through the increasing volumes of medical literature before making life critical decisions in time limited settings of the intensive care unit (ICU) [2].

**Fig. 1:**
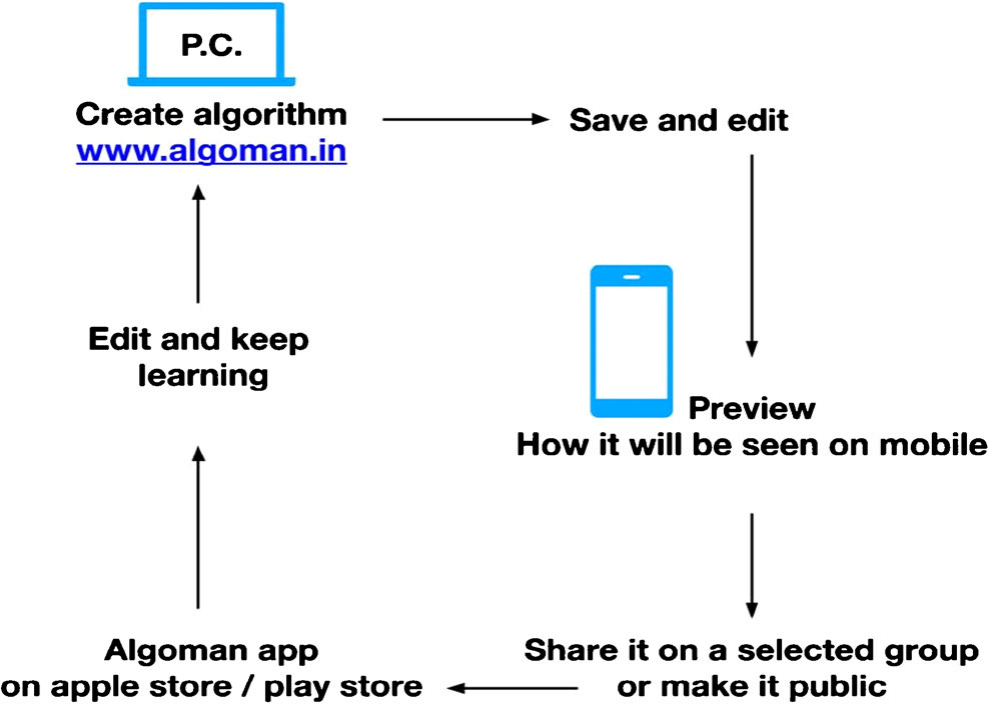
The working of an Algoman

The process of creating algorithm is as follows: After you login to the website, go to create algorithms. Click on the boxes, key in the text. The four dots on each of the box turn green when clicked, connect one dot to the other dot on another box by clicking on the latter. If you add step/ block, additional boxes get added, thus creating an algorithm (Fig. 1).

Algorithms have one major problem and that is they are vulnerable to changes and these are a corollary to ongoing research, for every change one needs to go through the process of coding and this means cost and time spent on going through the whole process repeatedly. Algoman is a simple solution to the complex problem of changing algorithms, and makes it easy to make an app on smartphones.

Computerized health diagnostics and decision making algorithms can provide timely clinical decision support at bedside and improvise the process of adherence to evidence based guidelines, and be a source for education and research [2]. Moreover, these algorithms can be integrated with hardware, and embedded with machine learning modules, to develop a fully functional artificial intelligence-based medico bot.

Thus, to conclude, Algoman can imbibe algorithmic learning which will be the future of medicine. Algorithms would create precision in diagnosis and management [4]. The future would be to add checklist to Algoman.
